# Assessing Public Health and Social Measures Against COVID-19 in Japan From March to June 2021

**DOI:** 10.3389/fmed.2022.937732

**Published:** 2022-07-12

**Authors:** Katsuma Hayashi, Taishi Kayano, Asami Anzai, Marie Fujimoto, Natalie Linton, Misaki Sasanami, Ayako Suzuki, Tetsuro Kobayashi, Kanako Otani, Masato Yamauchi, Motoi Suzuki, Hiroshi Nishiura

**Affiliations:** ^1^School of Public Health, Kyoto University, Kyoto, Japan; ^2^National Institute of Infectious Diseases, Tokyo, Japan

**Keywords:** coronavirus, statistical estimation, modeling, Infectious Disease, mathematical model, effective reproduction number

## Abstract

**Background:**

Public health and social measures (PHSM) against COVID-19 in Japan involve requesting the public to voluntarily reduce social contact; these measures are not legally binding. The effectiveness of such PHSM has been questioned with emergence of the SARS-CoV-2 Alpha variant (B.1.1.7), which exhibited elevated transmissibility.

**Materials and Methods:**

We investigated the epidemic dynamics during the fourth epidemic wave in Japan from March to June 2021 involving pre-emergency measures and declaration of a state of emergency (SoE). We estimated the effective reproduction number (*R*_*t*_) before and after these interventions, and then analyzed the relationship between lower *R*_*t*_ values and each PHSM.

**Results:**

With implementation of pre-emergency measures (PEM) in 16 prefectures, the *R*_*t*_ was estimated to be < 1 in six prefectures; its average relative reduction ranged from 2 to 19%. During the SoE, 8 of 10 prefectures had an estimated *R*_*t*_ < 1, and the average relative reduction was 26%–39%. No single intervention was identified that uniquely resulted in an *R*_*t*_ value < 1.

**Conclusion:**

An SoE can substantially reduce the *R*_*t*_ and may be required to curb a surge in cases caused by future SARS-CoV-2 variants of concern with elevated transmissibility. More customized interventions did not reduce the *R*_*t*_ value to < 1 in this study, but that may be partly attributable to the greater transmissibility of the Alpha variant.

## Introduction

Public health and social measures (PHSM) against COVID-19 are non-specific countermeasures, previously referred to as non-pharmaceutical interventions, which have been implemented in many countries to control the pandemic. Whereas published studies report a strong overall epidemiological impact of lockdowns or PHSM in reducing cases (1–14), other studies suggest that lockdown itself may not be very effective if individual measures, such as wearing masks and restricting the use of restaurants and public facilities, are properly implemented (15, 16). Additional scientific evidence on the effectiveness of individual non-specific countermeasures is called for (e.g., school closures, bans on eating out and on large gatherings) (17–34). When the SARS-CoV-2 Alpha variant (B.1.1.7) with elevated transmissibility started to become widespread in Europe, strict lockdown measures were instated in European countries, which precluded an evaluation of each PHSM other than movement restriction of entire communities (35).

In Japan, PHSM have primarily comprised request-based nationwide cooperation in voluntarily reducing social contact and have not involved any legally binding penalties. The legal and administrative basis of such countermeasures is the state of emergency (SoE), which in Japan is declared based on the Act on Special Measures for Pandemic Influenza and New Infectious Diseases Preparedness and Response (2002) (36). In Japan, the first SoE was declared in April 2020 owing to a surge in cases during the first epidemic wave. Although the first SoE was lifted in May 2020, an SoE was declared twice, in January and April 2021, for municipalities in the Tokyo metropolitan area and the Kansai region owing to increasing pressure on health care services during the third and fourth epidemic waves. During the fourth wave, a larger number of people were infected with the Alpha variant (B.1.1.7), which has greater transmissibility than other previously circulating SARS-CoV-2 strains (37, 38). Because an SoE involves substantial adverse social and economic effects, pre-emergency measures (PEM) were devised as an alternative to an SoE. More customized countermeasures began from February 2021, and PEM were newly implemented based on the Revised Special Measures for Pandemic Influenza and New Infectious Diseases Preparedness and Response (2020), in the hopes of avoiding an SoE (39). In the PEM, the local governor of the target area can decide which sub-regional areas (e.g., cities, towns, and villages) will be subject to the countermeasures. PEM is a general term for interventions that do not involve restrictions on movement; instead, the countermeasures are more customized and focused on high-risk settings, including eating and drinking establishments that serve alcohol (40). That is, whereas SoE may be regarded as self-restrained contact reduction or a voluntary lockdown measure, PEM are more customized pre-lockdown policies that are expected to be implemented early in the epidemic and that represent more targeted intervention in high-risk settings only. Customized policies are in line with the scientific evidence that high-risk situations include drinking alcohol late at night and attending indoor gatherings, which sometimes trigger super-spreading events (41, 42).

During the fourth epidemic wave, an SoE and PEM were declared multiple times, targeting municipalities across Japan. However, the effectiveness of these countermeasures in controlling infections has yet to be evaluated, especially considering the spread of the highly transmissible Alpha variant (43). These countermeasures have been diverse. In areas of intervention, different series of countermeasures have been implemented, including a request for residents to refrain from unnecessary movement and restricting the use of public facilities. In particular, the content, intensity, and duration of PEM have not been uniform because local governments decide the details regarding countermeasures. Like an SoE, PEM are not legally binding. Thus, the effectiveness of these measures depends on the compliance of residents and employers in areas of intervention and how well contact can be avoided that could lead to infection. Objectively assessing the effectiveness of such countermeasures remains a scientific challenge.

With emergence of the Alpha variant, which led to a surge in COVID-19 cases close to the Christmas holiday season, many European countries immediately imposed strict lockdown policies and did not permit customized interventions to be implemented (44). In many prefectures of Japan, PEM with various customized interventions were implemented, which were subsequently followed by an SoE that enforced restrictions on free movement. The fourth wave involving the Alpha variant in Japan thus offers a unique opportunity to evaluate the effectiveness of such countermeasures. Considering that there may be additional opportunities to implement PHSM in the future (45), and complete reliance on pharmaceutical interventions and vaccination is not possible (46), it is vital to explicitly assess those interventions. Thus, in the present study, we aimed to estimate the effectiveness of PHSM in Japan.

## Materials and Methods

### Epidemiological Data and Interventions

The present study was focused on the fourth epidemic wave in Japan, from March 1st to June 30, 2021, involving more than 370,000 confirmed COVID-19 cases and 6000 confirmed deaths across Japan. COVID-19 cases in Japan are confirmed by means of reverse transcription polymerase chain reaction (RT-PCR) and antigen testing, and all diagnosed cases are mandatorily notified to the government via the local health center. During the study period, two types of antigen detection kits using immunochromatography were approved in Japan, but PCR testing with a Taqman probe was used consistently for all confirmatory diagnosis (47). We used the incidence of confirmed cases as a function of the date of diagnosis and the date of illness onset as registered in the Health Center Real-Time Information-sharing System on COVID-19 (HER-SYS) (48). During the study period, random sampling of PCR-positive patients in Japan was conducted to screen for the N501Y point mutation that was commonly seen in the Alpha variant (B.1.1.7) using real time RT-PCR, so as to estimate the percentage of infections with this variant (49). As of March 28, approximately 30% of all infected patients identified on each day were screened (50). Some were tested using whole-genome sequencing, but the sequencing results were not used to determine the proportion of variants, because it takes about two weeks to report and cannot provide a timely picture of the infection situation in real time. The results of these screenings were entered into the HER-SYS along with other epidemiological information. We estimated the number of infections with the Alpha variant in the country according to the number of infected persons per day, the number of screening tests per day, and the number of cases that were positive for the N501Y point mutation.

Information regarding the types and length of PHSM were systematically collected according to prefecture and local government. According to the type of intervention, PHSM were classified into seven different categories: (a) official requests not to sell and serve alcoholic beverages and shortened business hours for restaurants at night; (b) closure of public facilities where large gatherings of people could be expected; (c) stay-at-home measures, combined with requests of the public not to travel across prefectures; (d) school closures; (e) requests to not organize public events; (f) requests to not engage in free movement within a city/ward; and (g) enlarging of the geographic area in which the above six countermeasures were implemented. To understand the impact of the epidemiological “stage” on reducing the effective reproduction number (*R*_*t*_), the following datasets were also collected and examined in relation to *R*_*t*_: hospital bed occupancy (both in general wards and intensive care units), the daily number of newly reported cases, daily PCR-positivity rate, which is the number of reported PCR-positive patients divided by the total number of PCR tests (51), and the proportion of unlinked infections among confirmed infections (i.e., the proportion of cases whose source could not be identified) (52). We used these variables to determine the epidemiological stage in Japan, leading to different levels of PHSM.

### Estimation of the Effective Reproduction Number

We used methods proposed by Nakajo and Nishiura for calculating *R*_*t*_ (53, 54). The *R*_*t*_ of COVID-19 was estimated as the epidemiological outcome, particularly its absolute and relative changes before and after the start of interventions. *R*_*t*_ was estimated using the incidence according to the date of illness onset (21). Letting *c*_*t*_ be the incidence according to date of illness onset *t*,


(1) $$E\left(c_{t}\right)=\sum_{\tau =0}^{t}R_{t-\tau }f_{\tau }\sum_{v=0}^{t-\tau +x}c_{t-\tau +x-v}\lambda_{v-x}$$

where E(.) is the expectation, *f*_*s*_ is the probability mass function of the incubation period duration *s*, and λ_*u*_ is the probability mass function of secondary transmission as a function of the time since illness onset *u*. The maximum likelihood method was used for the estimation of *R*_*t*_. It should be noted that the resulting estimated *R*_*t*_ is a function of the date of infection.

Following Nakajo and Nishiura (53, 54) we assumed that *x* is the duration of infectiousness prior to illness onset, and we set *x* = 5 days (i.e., cases became infectious 5 days prior to the illness onset date). *f*_*s*_ was assumed to follow a lognormal distribution with mean 5.2 days and variance 14.9 (55), and λ_*u*_ was assumed to follow a gamma distribution with mean 12.9 days and variance 8.1 (56).

The abovementioned equation was further applied to estimate the *R*_*t*_ for the Alpha variant only. For this, we reconstructed the incidence of infections with variant Alpha using the partially screened dataset of real time RT-PCR screening of cases for the N501Y mutation. As proposed by Murayama et al. (57), a hypergeometric distribution was used to estimate the incidence, i.e., on each day, the total number of cases infected with variant Alpha represents a random selection of screening tests conducted among the total number of confirmed cases and a random selection of cases that were positive for the Alpha variant among all screened cases.

The effectiveness of PEM or SoE was calculated by comparing the change in *R*_*t*_, that is, between (i) the *R*_*t*_ value 7 or 14 days prior to implementation of the PHSM and (ii) the first 7 days or total days of the PHSM. To facilitate this estimation, we used a piecewise constant model for *R*_*t*_ (i.e., handling *R*_*t*_ as a step function) for the respective periods. A 7-day period was specifically chosen for period (ii) because that period was followed by the so-called Golden Week, a spring holiday period in Japan of more than 10 consecutive days, which influences people’s mobility characteristics. In addition to relative and absolute risk reductions in secondary transmission, we also explored whether the *R*_*t*_ value during each intervention period (with PEM or SoE) was < 1, indicating that the incidence of COVID-19 infection was in a declining trend. We truncated the last 10 days of data and conducted analysis through May 27, 2021 because the most recent *R*_*t*_ estimation using onset data is an underestimate owing to reporting delay. We varied the combinations of duration of periods (i) and (ii) to assess the reductions in *R*_*t*_; for example, we alternatively used the estimated *R*_*t*_ for the entire period of intervention rather than using a fixed length of 7 days as part of sensitivity analysis. Among prefectures in which interventions were implemented, Miyagi was excluded from our analysis because most COVID-19 cases in Miyagi were caused by a SARS-CoV-2 strain with the E484K mutation, which was not classified as either a variant of concern or variant of interest (23). To avoid underestimation of *R*_*t*_, the dataset for the period under PEM in Hokkaido was analyzed for only 6 days post intervention; similarly, the dataset under the SoE in Okayama was analyzed for the first 5 days of intervention owing to right truncation. In Hokkaido, Gifu, and Mie prefectures, the 10-day national holiday period overlapped with the 7-day period immediately before the implementation of PEM, potentially overestimating their effectiveness. Thus, we also compared the *R*_*t*_ values before and after intervention, using the 7-day period before the start of the national holiday as an alternative baseline.

To explore the statistical association between a decline in *R*_*t*_ value and each individual countermeasure, we performed the Wilcoxon signed-rank test. Likewise, we carried out univariate analysis to explore the relationship between the stage of the epidemic when PHSM were implemented and a reduction in the *R*_*t*_ value.

## Results

### Changes in the Effective Reproduction Number

**FIGURE 1:**
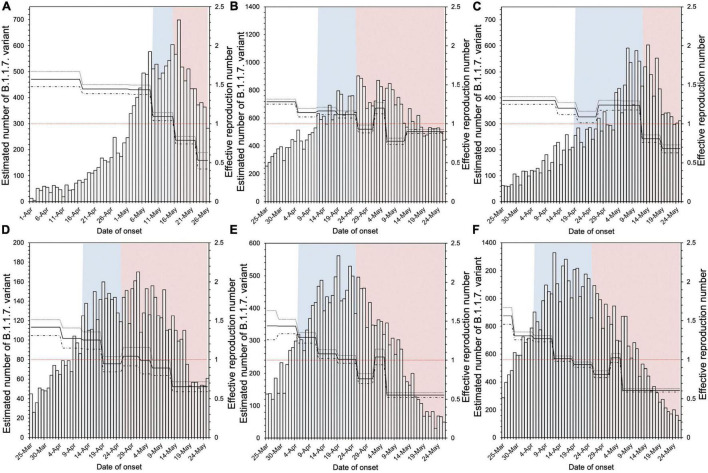
Estimated effective reproduction number in six prefectures implementing both pre-emergency measures and a state of emergency. **(A–F)** in the figure correspond to Hokkaido, Tokyo, Aichi, Kyoto, Osaka, and Hyogo prefectures, respectively. The blue shaded areas indicate the period of pre-emergency measures, and the red shaded areas indicate the state of emergency period. The bar graph is the estimated number of infections with the B.1.1.7 variant by onset date, and the solid line is the effective reproduction number (*R*_*t*_). Using the 7 days before implementation of pre-emergency measures as the baseline, we compared the *R*_*t*_ for the 7 days immediately after the start of each measure.

Figure 1 shows the *R*_*t*_ in six prefectures where both PEM and an SoE were implemented, using a step function for the first 7 days before and during intervention. Overall, a decreasing trend in the *R*_*t*_ was noted during the countermeasure period.

The relative reduction in *R*_*t*_ values after PEM implementation was estimated to range from −110.9% to 43.0% (Table 1), where negative values represent a failure to reduce the *R*_*t*_ value. Among a total of 16 prefectures following PEM, six prefectures achieved an *R*_*t*_ value < 1 (Gunma, Gifu, Mie, Ehime, Kumamoto, and Okinawa). The average and median relative reduction in *R*_*t*_ in these 16 prefectures was 2.0% and 9.0%, respectively. Supplementary Table 1 shows the relative reduction in *R*_*t*_, analyzing the values for the entire period of PEM implementation. The relative reduction in *R*_*t*_ was estimated to range from −15.9 to 51.6%, and the average and median relative reduction in *R*_*t*_ was estimated to be 11.3% and 14.8%, respectively, among a total of 16 prefectures. Using the *R*_*t*_ of the 14 days prior to PEM implementation, the relative reduction in *R*_*t*_ was estimated to range from −18.5 to 49.7% and the average and median relative reduction in *R*_*t*_ was estimated to be 19.4% and 20.6%, respectively.

**TABLE 1:** Effective reproduction number (*R*_*t*_) during the 7 days before and after pre-emergency measures (PEM) were instated.

Prefecture	Average Rt during the 7 days pre-PEM instatement	Average Rt during the 7 days post-PEM instatement	Absolute reduction in *R*_*t*_	Relative reduction in *R*_*t*_
Hokkaido*	1.44 (1.38, 1.50)	1.09 (1.04, 1.14)	0.34 (0.26, 0.45)	0.24 (0.19, 0.30)
Gunma	0.66 (0.54, 0.79)	0.95 (0.78, 1.10)	−0.30 (−0.49, −0.03)	−0.45 (−0.86, −0.03)
Saitama	1.19 (1.11, 1.29)	1.09 (0.99, 1.17)	0.11 (−0.04, 0.26)	0.09 (−0.03, 0.21)
Chiba	1.11 (1.00, 1.21)	1.10 (0.98, 1.20)	0.01 (−0.15, 0.23)	0.01 (−0.15, 0.19)
Tokyo	1.14 (1.09, 1.20)	1.16 (1.11, 1.21)	−0.02 (−0.10, 0.07)	−0.02 (−0.09, 0.05)
Kanagawa	1.11 (1.03, 1.19)	1.08 (0.98, 1.16)	0.03 (−0.10, 0.18)	0.03 (−0.09, 0.15)
Ishikawa	0.65 (0.46, 0.83)	1.37 (1.07, 1.55)	−0.72 (−0.93, −0.29)	−1.11 (−1.89, −0.37)
Gifu*	1.41 (1.28, 1.52)	0.80 (0.70, 0.89)	0.61 (0.43, 0.80)	0.43 (0.33, 0.53)
Aichi	1.20 (1.12, 1.27)	1.09 (1.01, 1.15)	0.11 (0.00, 0.24)	0.09 (0.00, 0.19)
Mie*	1.19 (1.01, 1.37)	0.83 (0.64, 0.99)	0.35 (0.11, 0.70)	0.30 (0.10, 0.52)
Kyoto	1.27 (1.14, 1.40)	1.25 (1.13, 1.36)	0.02 (−0.16, 0.24)	0.02 (−0.13, 0.17)
Osaka	1.30 (1.25, 1.36)	1.27 (1.23, 1.31)	0.04 (−0.04, 0.12)	0.03 (−0.03, 0.09)
Hyogo	1.44 (1.35, 1.52)	1.29 (1.22, 1.36)	0.14 (0.02, 0.27)	0.10 (0.02, 0.18)
Ehime	0.72 (0.53, 0.92)	0.64 (0.34, 0.87)	0.08 (−0.24, 0.54)	0.11 (−0.43, 0.61)
Kumamoto	0.80 (0.69, 0.92)	0.58 (0.42, 0.70)	0.23 (0.05, 0.46)	0.28 (0.07, 0.52)
Okinawa	1.12 (0.98, 1.24)	0.92 (0.80, 1.02)	0.20 (0.02, 0.41)	0.18 (0.02, 0.33)

*Values in parentheses are bootstrapped 95% confidence intervals.*

*The 7-day period prior to implementation of pre-emergency measures in Hokkaido, Gifu, and Mie prefectures overlapped with a 10-day national holiday. Considering the 7-day period before the start of the holiday period instead (to eliminate the influence of holiday mobility on R_t_), the relative reduction in R_t_ was estimated to be 0.21 (0.15, 0.28) in Hokkaido, 0.44 (0.34, 0.53) in Gifu, and −0.02 (−0.29, 0.27) in Mie.

Similar analysis was conducted for the SoE (Table 2). Among the 10 prefectures under an SoE, the relative reduction in *R*_*t*_ values under the SoE was estimated to range from −13.4 to 47.1%, using the 7-day average *R*_*t*_ before intervention as the baseline. The average and median relative reduction in *R*_*t*_ was estimated to be 25.9% and 31.5%, respectively. Kyoto and Okinawa did not achieve an *R*_*t*_ < 1 within 7 days of the start of the SoE; however, the *R*_*t*_ in the remaining eight prefectures was estimated to be < 1. Supplementary Table 2 shows the results for the SoE in different comparison periods before and after the intervention. The relative reduction in *R*_*t*_ under the SoE was estimated to range from −28.2 to 52.7%, using the 7-day average *R*_*t*_ before the intervention as the baseline. The average and median relative reduction in *R*_*t*_ was estimated to be 27.6% and 37.7%, respectively. Using the *R*_*t*_ of the 14 days before the start of the SoE, the relative reduction in *R*_*t*_ was estimated to range from −18.5 to 61.0%, and the median relative reduction in *R*_*t*_ was estimated to be 38.7% and 48.6%, respectively.

**TABLE 2:** Effective reproduction number (*R*_*t*_) during the 7 days before and after declaration of a state of emergency (SoE).

Prefecture	Average Rt during the 7 days pre-SoE declaration	Average Rt during the 7 days post-SoE declaration	Absolute reduction in *R*_*t*_	Relative reduction in *R*_*t*_
Hokkaido*	1.44 (1.38, 1.50)	0.79 (0.74, 0.84)	0.65 (0.58, 0.72)	0.45 (0.41, 0.49)
Tokyo	1.14 (1.09, 1.20)	0.93 (0.88, 0.98)	0.21 (0.14, 0.29)	0.19 (0.13, 0.25)
Aichi	1.20 (1.12, 1.27)	0.81 (0.76, 0.86)	0.39 (0.29, 0.49)	0.33 (0.26, 0.39)
Kyoto	1.27 (1.14, 1.40)	1.04 (0.92, 1.16)	0.23 (0.07, 0.42)	0.18 (0.06, 0.31)
Osaka	1.30 (1.25, 1.36)	0.81 (0.77, 0.86)	0.49 (0.42, 0.56)	0.38 (0.33, 0.42)
Hyogo	1.44 (1.35, 1.52)	0.76 (0.70, 0.83)	0.67 (0.57, 0.78)	0.47 (0.41, 0.52)
Okayama	0.48 (0.39, 0.56)	0.54 (0.41, 0.64)	−0.06 (−0.21, 0.14)	−0.13 (−0.51, 0.26)
Hiroshima	0.97 (0.90, 1.06)	0.68 (0.59, 0.75)	0.30 (0.16, 0.44)	0.31 (0.18, 0.43)
Fukuoka	1.09 (1.03, 1.14)	0.58 (0.52, 0.63)	0.51 (0.42, 0.60)	0.47 (0.40, 0.53)
Okinawa	1.12 (0.98, 1.24)	1.16 (1.04, 1.26)	−0.04 (−0.20, 0.13)	−0.04 (−0.19, 0.11)

*Values in parentheses are bootstrapped 95% confidence intervals.*

*The 7-day pre-SoE period in Hokkaido overlapped with a 10-day national holiday. Considering the 7-day period before the start of the holiday period instead (to eliminate the influence of holiday mobility on the R_t_), the relative reduction in R_t_ was estimated to be 0.44 (0.38, 0.49).

### Content and Timing of Interventions

**FIGURE 2:**
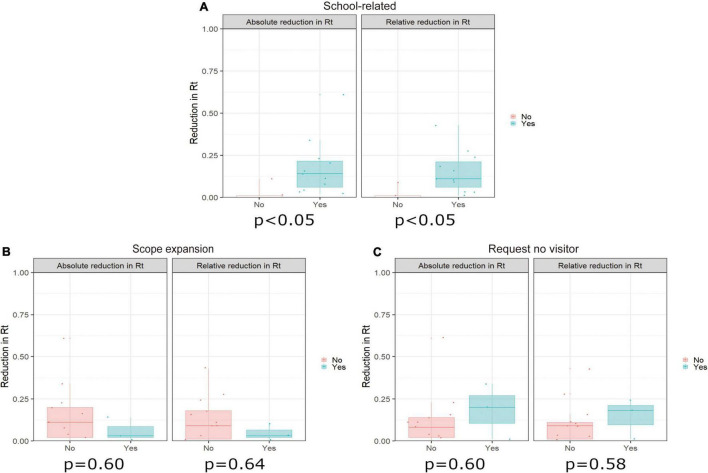
Relationship between pre-emergency measure (PEM) categories and reduction in the effective reproduction number (*R*_*t*_) compared with the 7 days before intervention (baseline period). This figure shows the relationship between the reduction in *R*_*t*_ during the 7 days prior to the intervention and during the 7 days after the intervention and presence of each PEM intervention. The only PEM categories for which there were differences in adoption among prefectures were **(A)** school-related measures, **(B)** expansion of the intervention scope (a pre-emergency intervention originally implemented in only part of a given prefecture was expanded to additional areas), and **(C)** requests (from the prefecture) for no out-of-prefecture travel. We calculated p-values using the Wilcoxon signed-rank test. The left-hand panel in each figure shows the absolute reduction in *R*_*t*_ and the right-hand panel shows the relative reduction in *R*_*t*_.

Figure 2 shows distributions of the absolute reduction and relative reduction in *R*_*t*_ values, grouped according to content of the PEM. The reduction in *R*_*t*_ tended to be greater in prefectures implementing school-related interventions than in the remaining prefectures. Interventions associated with eating and drinking establishments, recreational and community facilities, and large gatherings or events were implemented in all prefectures during the PEM period; therefore, we were unable to make comparisons for these measures.

Supplementary Figure 1 shows results regarding the SoE, which were similar to those of the PEM. We did not identify any significant differences regarding the reduction in *R*_*t*_ according to different types of intervention. These findings were consistent across different durations of baseline and intervention periods.

**FIGURE 3:**
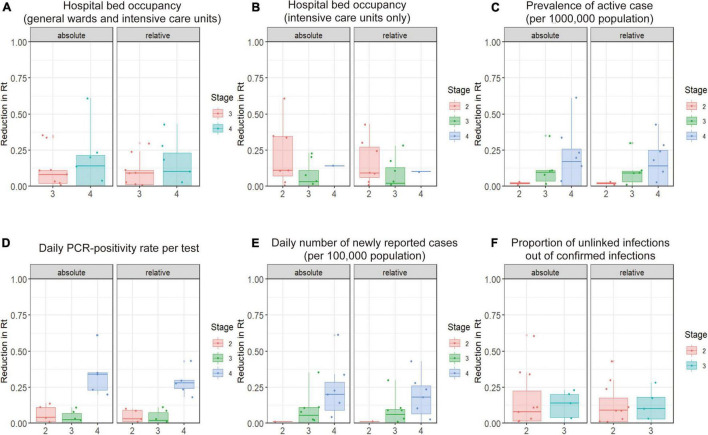
Relationship between prefectural COVID-19 epidemic stage at the start of pre-emergency measures (PEM) and reduction in the effective reproduction number (*R*_*t*_) during the PEM period compared with the 7 days before intervention (baseline period). This figure shows the relationship between the reduction in *R*_*t*_ during the 7 days prior to the intervention and during the 7 days after the intervention and the epidemic stage at the time of the intervention. The horizontal axis is the “stage” of the COVID-19 epidemic, according to definitions of the Japanese government. **(A,B)** Hospital-bed occupancy is defined as stage 3 when 20% of COVID-19 beds are occupied and stage 4 when 50% of beds are occupied. **(C)** The prevalence of active cases is defined as the number of patients who are hospitalized or under observation at home. Twenty or more cases per 100,000 population in a prefecture is defined as stage 3, and 30 or more cases is defined as stage 4. **(D)** The daily PCR-positivity rate is defined as stage 3 with 5% or more positive test results among the total tests and stage 4 with 10% or more. **(E)** The daily number of newly reported cases is defined as stage 3 with 15 or more newly reported cases per 100,000 population and stage 4 with 25 or more newly reported cases per 100,000. **(F)** The percentage of unlinked cases is defined as 50% or more for stage 3 and less than 50% for stage 2. The left-hand panel shows the absolute reduction in the effective reproduction number (*R*_*t*_) and the right-hand panel shows the relative reduction. We calculated p-values using analysis of variance or the Wilcoxon signed-rank test.

Figure 3 illustrates distributions of the absolute reduction and relative reduction in *R*_*t*_ according to the stage of the epidemic when interventions were carried out (as classified into four categories; see Figure 3 legend for the definitions). The reduction in *R*_*t*_ values in stage 4 of the PCR positivity rate tended to be greater than that in stages 2 and 3; the daily PCR positivity rate is defined here as: 5% or more of all tests positive is stage 3 and 10% or more is stage 4. Supplementary Figure 2 shows the results of analysis of stages at the time of SoE implementation. No marked association was identified between the stage of the epidemic and relative reduction in *R*_*t*_ during the SoE. This finding was maintained across varying durations of the baseline and intervention periods.

## Discussion

In the present study, we evaluated the impact of the PEM and SoE on the epidemiological dynamics of COVID-19 from March to June 2021 in different regions of Japan, during which time the SARS-CoV-2 Alpha variant was predominant in the country. When PEM were implemented in 16 prefectures, the *R*_*t*_ was reduced to < 1 in only six of these prefectures, with the average relative reduction ranging from 2 to 19%. However, implementation of an SoE led to an average *R*_*t*_ value < 1 in 8 of the 10 prefectures where implemented, with an average relative reduction in the *R*_*t*_ ranging from 26 to 39%. For individual interventions, only school closures during periods of PEM implementation showed significant differences in reducing the *R*_*t*_; no other interventions helped to explain variations in the relative reduction in *R*_*t*_. Although there was no significant association between the relative reduction in *R*_*t*_ and the timing of initiating interventions, an extremely high positivity rate in PCR testing may predict a substantial reduction in the *R*_*t*_. An increasing trend over time in the positivity rate for PCR testing results has been shown to predict a forthcoming epidemic wave (58). The present study findings further showed that PCR testing results that are extremely high, i.e., a very high positivity rate, may be one signature of a forthcoming reduction in the *R*_*t*_, perhaps because a very high positivity rate is usually a sign of the need for strong intervention measures.

The findings of the present study revealed is that customized interventions (referred to as PEM in our study) in high-risk transmission settings may be insufficient to lower the *R*_*t*_ to < 1. Before the introduction of variant Alpha in Japan, a simple modeling study showed that *R*_*t*_ decreased to < 1 only after instating customized PHSM focused on high-risk settings. During the summer of 2020, interventions were centered on eating and drinking establishments operating at night and public facilities for mass gatherings and included shortening of opening hours, requests to not serve alcohol in Tokyo, restricting the number of people eating at the same table to four, and closure of nightlife areas in Osaka (59). Through that experience, such focused interventions were legally formalized by the Japanese government and categorized as PEM to help avoid unnecessary adverse social and economic impacts on the entire population. Unfortunately, with introduction of the more-transmissible Alpha variant, our findings showed that in many prefectures, the introduction of PEM alone was not sufficient to reduce the *R*_*t*_ value to below 1 during the spring of 2021. From July 2021, the SARS-CoV-2 Delta variant, which is even more transmissible than variant Alpha (60, 61), was introduced and rapidly became predominant among all virus strains circulating in Japan (62). Whereas elevated temperatures may help to slightly lower the *R*_*t*_ value (63), only stronger restrictions such as an SoE can suppress a sharp rise in COVID-19 infections (64, 65). Therefore, swift decisions regarding declaration of an SoE are required to bring an epidemic under control.

The PHSM explored in this present study are not accompanied by legal penalties In Japan; instead, both PEM and an SoE involve an official request from national and local governmental bodies to adhere to the policies (36). Furthermore, even without government intervention, people may precautionarily adopt risk-avoidance behavior, especially when the number of infected individuals is increased. Because the PEM and SoE were not legally binding in principle, we cannot explicitly determine whether people actually reduced their risky behaviors voluntarily (but actively) under the interventions (66). This characteristic complicates the evaluation in two ways. First, we may not always be able to anticipate whether public reactions and behavioral responses to PEM or an SoE will be the same as those observed from March to June 2021. If the general public has difficulty enduring restrictions on high-risk behaviors, the findings of the present study may not be applicable and cannot be expected in the future (67). In this sense, request-based (voluntary) restriction of social contact behaviors cannot be causally evaluated and the effectiveness of repeated implementation cannot be ensured. Second, psychological impacts could influence our outcomes. The declaration of an SoE itself might have had the effect of preventing risky behaviors, but such behavioral changes might have been induced primarily by elevated risk awareness, e.g., the declaration led to voluntary cancelation of travel and large gatherings or events (68). As such, it is vital to remember that the PEM and SoE in Japan rely on voluntary cooperation of the general public and employers, and people’s psychological responses to such requests have a key role in the effect of intervention. In these respects, it is inherently difficult to separate the effects of intervention from the effects of voluntary risk-avoidance behaviors. We performed additional analyses with the aim to strengthen our findings. As shown in Supplementary Table 3, we made a comparison of the *R*_*t*_ at 1 and 2 weeks prior to the implementation of PEM. Indeed, whereas the *R*_*t*_ decreased before the start of PEM in some prefectures and this decrease was large, the same trend was not observed in many prefectures. Supplementary Figure 3 depicts the change in Google mobility before and after intervention. Although in some prefectures, mobility had already decreased before the PEM, it was found that mobility generally decreased with the intervention. These supplementary analyses do not refute the effect of voluntary risk-avoidance behaviors. Rather, the important point here is that many prefectures were unable to reduce the *R*_*t*_ to below 1 with customized PEM that mostly targeted eating and drinking establishments whereas the SoE was able to flatten the epidemic curve.

It is worth mentioning that in a part of our analysis, we adopted a baseline period of the 7 days prior to the implementation of PHSM. Of course, risk awareness had been gradually increasing owing to the increasing epidemic size prior to the start of PHSM; therefore, people’s social contact behavior could have already begun to change during the 7-day baseline period. Thus, as part of the sensitivity analysis, the duration of the baseline period was altered. Comparing the *R*_*t*_ 7 days prior to the intervention and during the entire intervention period, PEM and the SoE reduced the *R*_*t*_ by an average of 11.3% and 27.6%, respectively. When the baseline was set to 14 days, the PEM and SoE decreased the *R*_*t*_ by an average of 19.4% and 38.7%, respectively. However, this analysis did not substantially alter our findings.

Our results regarding school closures during PEM were consistent with those of published studies (69–74). School closures during an SoE did not lead to a marked reduction in *R*_*t*_; therefore, school closures would not be consistently effective across all possible epidemiological conditions (74, 75).

Caution is needed when discussing the timing of PHSM implementation (74). The short-term goals of PHSM may include to (i) suppress an epidemic, (ii) ease caseload pressure on health care facilities, and (iii) buy time to increase protection via pharmaceutical interventions (e.g., vaccination). If goal (iii) is not critical, early implementation of PHSM will always be the most effective owing to the containment of an epidemic at a local level. This is particularly true for PEM; the risk of an epidemic wave could have been greatly reduced if PEM had been implemented before a substantial increase in the COVID-19 incidence and if the PEM covered the areas or regions with increasing virus transmission. It must be remembered that the estimated relative reduction in *R*_*t*_ found in the present study is conditional on implementation at a given timing; in general, the effectiveness of PHSM is greater if implemented earlier (76, 77).

Certain limitations in this study must be acknowledged. First, the quantified risk reduction would not be causal, as mentioned above; thus, similar impacts of PHSM are not guaranteed (40). Second, our analysis relied on the change in *R*_*t*_ before and after intervention. We argued that this change may be attributable to extrinsic effects, namely, PHSM. However, other extrinsic factors (e.g., behavioral changes) and intrinsic factors (e.g., depletion of susceptibles) can also lead to a reduction in the *R*_*t*_ value. In fact, there were continual government announcements with respect to the ongoing risk of infection and requests to wear a face mask, maintain an appropriate social distance, and engage in preventive measures (76). Third, in our analysis, we did not evaluate the time-dependent variations in intervention effectiveness. During the later stages of PHSM, the degree of preventive effectiveness can sometimes be magnified, perhaps owing to a gradual reduction in high-risk contacts. Fourth, we used the prefecture as the unit in our analysis, and these were analyzed independently. Spatial correlations associated with travel across prefectures could not be controlled (40).

Although not causal, the present study provides important evidence indicating that achieving a substantial reduction in *R*_*t*_ in the presence of a highly transmissible SARS-CoV-2 variant requires implementation of an SoE. Continued epidemiological assessment of PHSM is critical, alongside further analysis of the heterogeneities in effectiveness among interventions.

## Data Availability Statement

The original contributions presented in the study are included in the article/Supplementary Material, further inquiries can be directed to the corresponding author.

## Ethics Statement

This study was approved by the Medical Ethics Board of the Graduate School of Medicine at Kyoto University (R2676).

## Author Contributions

KH conceived the study. KH and HN conceptualized the study design and drafted early versions of the manuscript and figures. KH, AA, MF, AS, TK, and MS collected the data. KH, NL, AA, MF, AS, and TK performed the statistical analyses. All authors provided comments on the manuscript and approved the final version of the manuscript.

## Conflict of Interest

The authors declare that the research was conducted in the absence of any commercial or financial relationships that could be construed as a potential conflict of interest.

## Publisher’s Note

All claims expressed in this article are solely those of the authors and do not necessarily represent those of their affiliated organizations, or those of the publisher, the editors and the reviewers. Any product that may be evaluated in this article, or claim that may be made by its manufacturer, is not guaranteed or endorsed by the publisher.

## Supplementary Material

Supplementary Figure 1 Relationship between state of emergency (SoE) categories and reduction in the effective reproduction number (*R*_*t*_) during an SoE compared with the 7 days before intervention (baseline period). This figure shows the relationship between the reduction in ***R*_*t*_** during the 7 days prior to the intervention and during the 7 days after the intervention and presence of each SoE intervention. The only SoE categories for which there were differences in adoption among prefectures were **(A)** school-related measures, **(B)** expansion of the intervention scope (SoE originally implemented in only part of a given prefecture expanded to additional areas), and **(C)** requests (from the prefecture) for no out-of-prefecture travel. We calculated p-values using the Wilcoxon signed-rank test. The left-hand panel in each figure shows the absolute reduction in *R*_*t*_ and the right-hand panel shows the relative reduction in *R*_*t*_.

Supplementary Figure 2 Relationship between prefectural COVID-19 epidemic stage at the start of a state of emergency (SoE) and reduction in the effective reproduction number (*R*_*t*_) during the SoE compared with the 7 days before intervention (baseline period). This figure shows the relationship between the reduction in ***R*_*t*_** during the 7 days prior to the intervention and during the 7 days after the intervention and the epidemic stage at the time of the intervention. The horizontal axis is the “stage” of the COVID-19 epidemic according to definitions of the Japanese government. **(A,B)** Hospital-bed occupancy is defined as stage 3 when 20% of COVID-19 beds are occupied and stage 4 when 50% of beds are occupied. **(C)** The prevalence of active cases is defined as the number of patients who are hospitalized or under observation at home. Twenty or more cases per 100,000 population in a prefecture is defined as stage 3, and 30 or more cases is defined as stage 4. **(D)** The daily PCR-positivity rate is defined as stage 3 with 5% or more positive test results among the total tests and stage 4 with 10% or more. **(E)** The daily number of newly reported cases is defined as stage 3 with 15 or more newly reported cases per 100,000 population and stage 4 with 25 or more newly reported cases per 100,000. **(F)** The percentage of unlinked cases is defined as 50% or more for stage 3 and less than 50% for stage 2. The left-hand panel shows the absolute reduction in the effective reproduction number (*R*_*t*_) and the right-hand panel shows the relative reduction. We calculated p-values using analysis of variance or the Wilcoxon signed-rank test.

Supplementary Figure 3 Google mobility: retail and recreation percent change from baseline during and before intervention. The broken line shows the retail and recreation percent change from baseline for mobility provided by Google. The relative change in time spent compared with that at the baseline of January 2020 is shown. The blue shading in the figure indicates pre-emergency measures (PEM), the red shading indicates state of emergency (SoE), the yellow shading indicates PEM 1 week earlier, and the green shading indicates PEM 2 weeks earlier. Panels **(A–F)** in the figure correspond to Hokkaido, Tokyo, Aichi, Kyoto, Osaka, and Hyogo prefectures, respectively. In **(A,D)**, mobility appears to have already started decreasing before the intervention. In the other panels, the effect of intervention appears to be working, especially for the SoE.
