# Supplementary figures and images for: Assessing Public Health and Social Measures Against COVID-19 in Japan From March to June 2021
Source: Front Med (Lausanne). 2022 Jul 12;9:937732. doi: 10.3389/fmed.2022.937732 (PMC9315273; doi:10.3389/fmed.2022.937732)

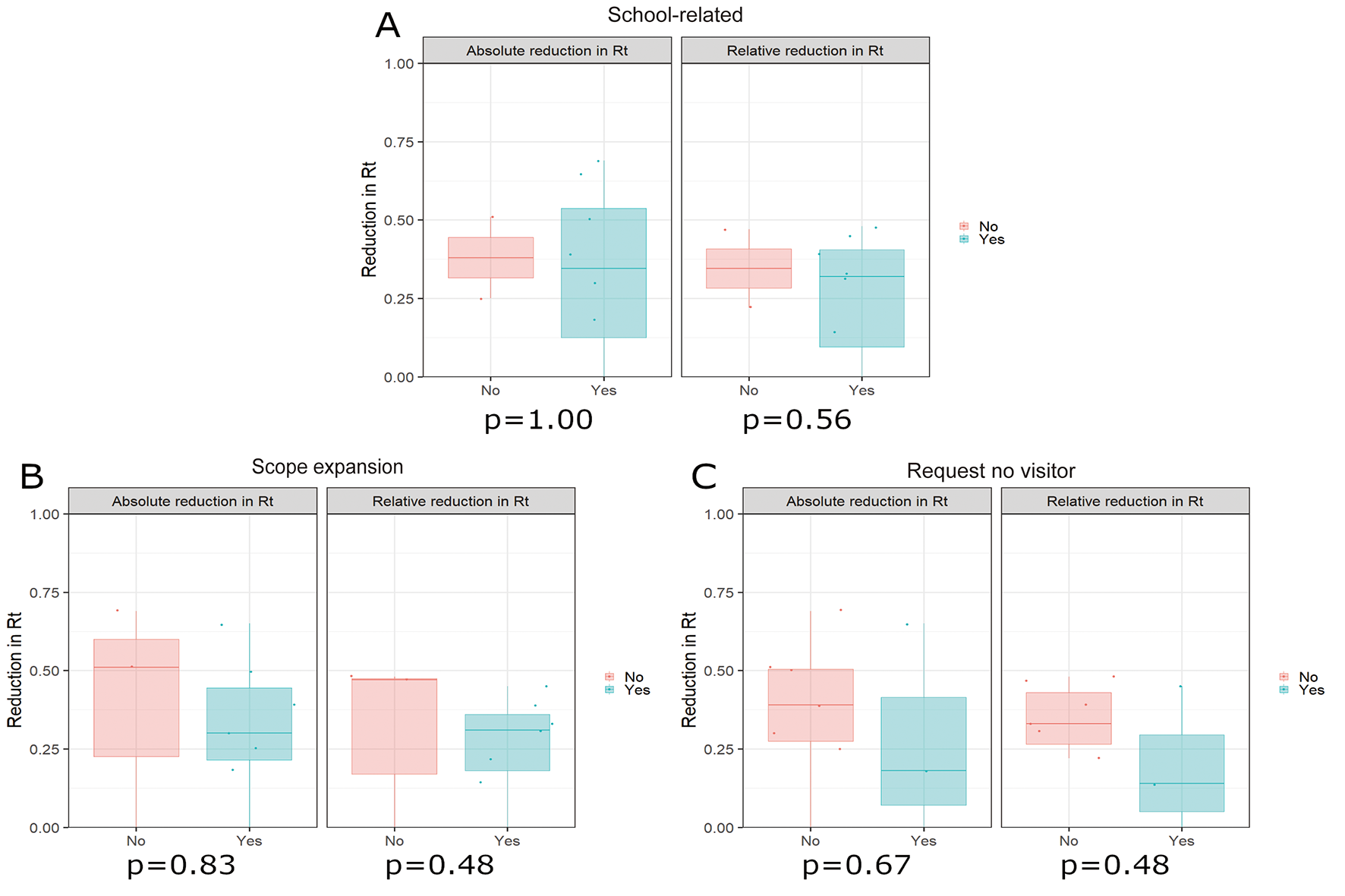

Supplement: Supplementary Figure 1 — Relationship between state of emergency (SoE) categories and reduction in the effective reproduction number (Rt) during an SoE compared with the 7 days before intervention (baseline period). This figure shows the relationship between the reduction in Rt during the 7 days prior to the intervention and during the 7 days after the intervention and presence of each SoE intervention. The only SoE categories for which there were differences in adoption among prefectures were (A) school-related measures, (B) expansion of the intervention scope (SoE originally implemented in only part of a given prefecture expanded to additional areas), and (C) requests (from the prefecture) for no out-of-prefecture travel. We calculated p-values using the Wilcoxon signed-rank test. The left-hand panel in each figure shows the absolute reduction in Rt and the right-hand panel shows the relative reduction in Rt. [file Image_1.TIF]

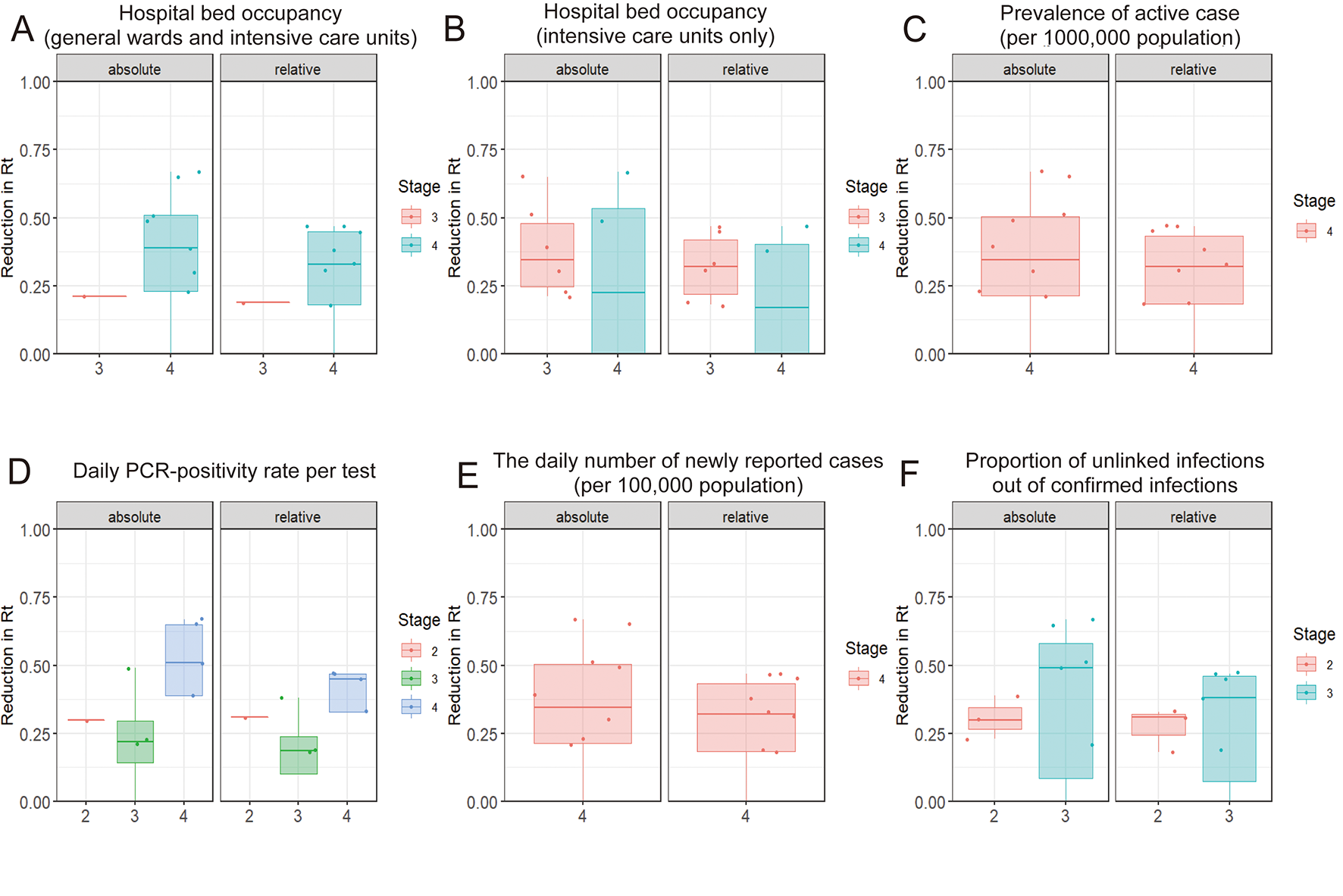

Supplement: Supplementary Figure 2 — Relationship between prefectural COVID-19 epidemic stage at the start of a state of emergency (SoE) and reduction in the effective reproduction number (Rt) during the SoE compared with the 7 days before intervention (baseline period). This figure shows the relationship between the reduction in Rt during the 7 days prior to the intervention and during the 7 days after the intervention and the epidemic stage at the time of the intervention. The horizontal axis is the “stage” of the COVID-19 epidemic according to definitions of the Japanese government. (A,B) Hospital-bed occupancy is defined as stage 3 when 20% of COVID-19 beds are occupied and stage 4 when 50% of beds are occupied. (C) The prevalence of active cases is defined as the number of patients who are hospitalized or under observation at home. Twenty or more cases per 100,000 population in a prefecture is defined as stage 3, and 30 or more cases is defined as stage 4. (D) The daily PCR-positivity rate is defined as stage 3 with 5% or more positive test results among the total tests and stage 4 with 10% or more. (E) The daily number of newly reported cases is defined as stage 3 with 15 or more newly reported cases per 100,000 population and stage 4 with 25 or more newly reported cases per 100,000. (F) The percentage of unlinked cases is defined as 50% or more for stage 3 and less than 50% for stage 2. The left-hand panel shows the absolute reduction in the effective reproduction number (Rt) and the right-hand panel shows the relative reduction. We calculated p-values using analysis of variance or the Wilcoxon signed-rank test. [file Image_2.TIF]

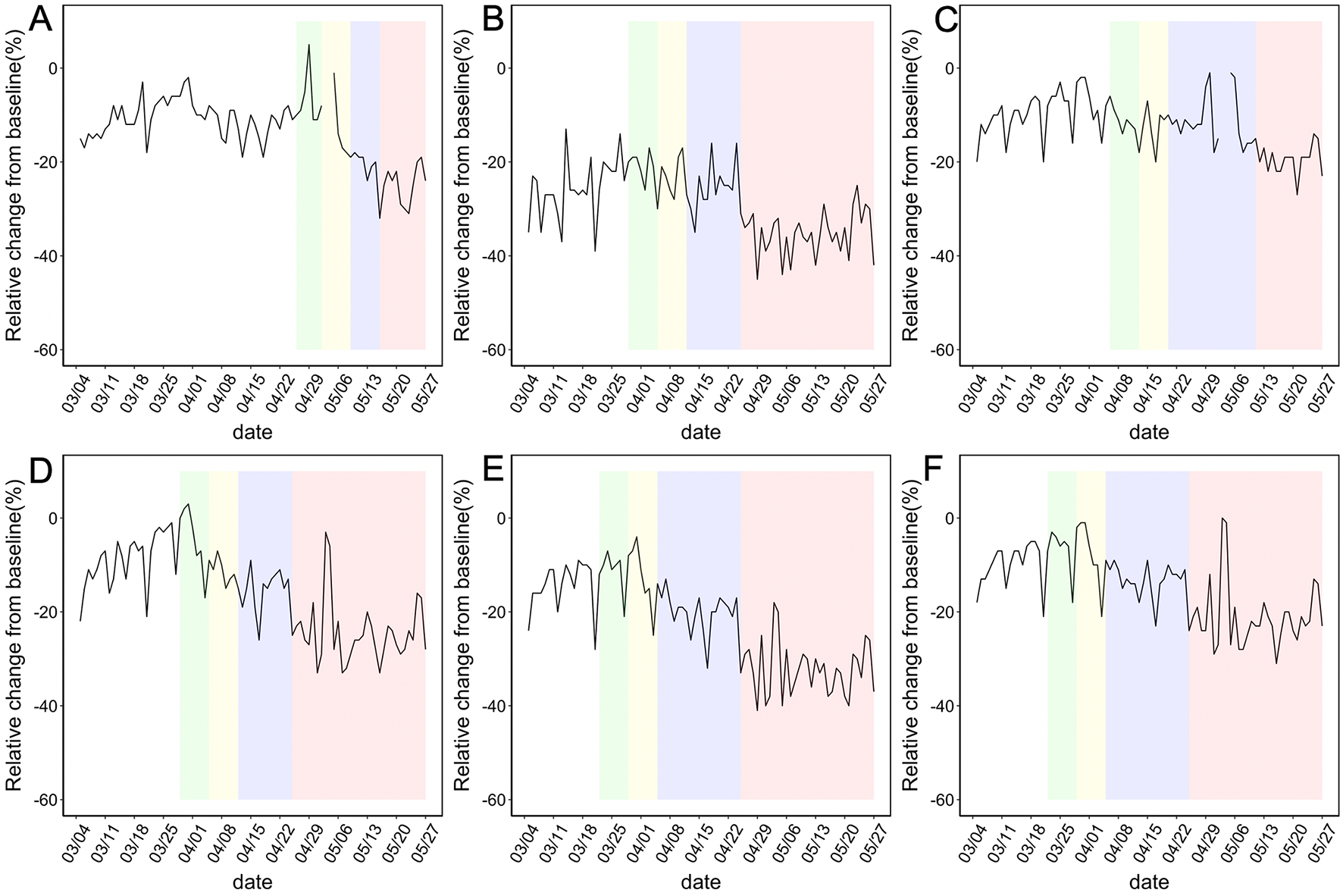

Supplement: Supplementary Figure 3 — Google mobility: retail and recreation percent change from baseline during and before intervention. The broken line shows the retail and recreation percent change from baseline for mobility provided by Google. The relative change in time spent compared with that at the baseline of January 2020 is shown. The blue shading in the figure indicates pre-emergency measures (PEM), the red shading indicates state of emergency (SoE), the yellow shading indicates PEM 1 week earlier, and the green shading indicates PEM 2 weeks earlier. Panels (A–F) in the figure correspond to Hokkaido, Tokyo, Aichi, Kyoto, Osaka, and Hyogo prefectures, respectively. In (A,D), mobility appears to have already started decreasing before the intervention. In the other panels, the effect of intervention appears to be working, especially for the SoE. [file Image_3.TIFF]
