# Supplementary material for: Assessing Public Health and Social Measures Against COVID-19 in Japan From March to June 2021
Source: Front Med (Lausanne). 2022 Jul 12;9:937732. doi: 10.3389/fmed.2022.937732 (PMC9315273; doi:10.3389/fmed.2022.937732)
Supplement: Supplementary file 4 [file Table_1.docx]

**Table S1 Reduction in the effective reproduction number (Rt), comparing a varying baseline period with the entire period of pre-emergency measure (PEM) implementation**

| Prefecture | Absolute reduction in Rt  (7 days pre-PEM vs entire PEM period) | Relative reduction in Rt  (7 days pre-PEM vs entire PEM period) | Absolute reduction in Rt  (14 days pre-PEM vs entire PEM period) | Relative reduction in Rt  (14 days pre-PEM vs entire PEM period) |
| --- | --- | --- | --- | --- |
| Hokkaido* | 0.30 (0.20, 0.40) | 0.21 (0.15, 0.27) | 0.31 (0.23, 0.40) | 0.21 (0.16, 0.27) |
| Gunma | 0.03 (-0.14, 0.24) | 0.04 (-0.22, 0.29) | 0.21 (0.09, 0.37) | 0.24 (0.11, 0.40) |
| Saitama | 0.31 (0.22, 0.40) | 0.25 (0.19, 0.30) | 0.31 (0.25, 0.38) | 0.25 (0.21, 0.29) |
| Chiba | 0.20 (0.10, 0.32) | 0.17 (0.09, 0.25) | 0.24 (0.16, 0.32) | 0.20 (0.14, 0.25) |
| Tokyo | -0.01 (-0.07, 0.06) | -0.01 (-0.06, 0.05) | 0.01 (-0.03, 0.06) | 0.01 (-0.03, 0.05) |
| Kanagawa | 0.14 (0.06, 0.23) | 0.12 (0.06, 0.19) | 0.21 (0.15, 0.27) | 0.17 (0.13, 0.22) |
| Ishikawa | -0.19 (-0.37, 0.10) | -0.25 (-0.59, 0.11) | 0.03 (-0.14, 0.20) | 0.03 (-0.15, 0.19) |
| Gifu* | 0.75 (0.62, 0.91) | 0.52 (0.46, 0.58) | 0.71 (0.60, 0.82) | 0.50 (0.45, 0.55) |
| Aichi | -0.05 (-0.13, 0.04) | -0.04 (-0.12, 0.04) | 0.02 (-0.03, 0.09) | 0.02 (-0.03, 0.07) |
| Mie* | 0.34 (0.14, 0.59) | 0.29 (0.13, 0.44) | 0.16 (0.02, 0.32) | 0.16 (0.02, 0.29) |
| Kyoto | 0.19 (0.04, 0.36) | 0.15 (0.04, 0.25) | 0.20 (0.10, 0.31) | 0.15 (0.08, 0.23) |
| Osaka | 0.32 (0.27, 0.37) | 0.23 (0.20, 0.26) | 0.41 (0.37, 0.45) | 0.28 (0.25, 0.30) |
| Hyogo | 0.37 (0.29, 0.47) | 0.25 (0.21, 0.30) | 0.39 (0.32, 0.45) | 0.26 (0.22, 0.29) |
| Ehime | -0.06 (-0.24, 0.21) | -0.09 (-0.44, 0.24) | 0.21 (0.06, 0.37) | 0.22 (0.08, 0.36) |
| Kumamoto | 0.14 (-0.02, 0.33) | 0.18 (-0.02, 0.36) | 0.53 (0.41, 0.67) | 0.48 (0.39, 0.58) |
| Okinawa | -0.16 (-0.27, -0.02) | -0.16 (-0.31, -0.02) | -0.07 (-0.16, 0.02) | -0.07 (-0.15, 0.01) |

Values in parentheses are bootstrapped 95% confidence intervals.

* The 7- and 14-day baseline periods preceding the implementation of PEM in Hokkaido, Gifu, and Mie prefectures overlapped with a 10-day national holiday. Considering the 7-day period before the start of the holiday period instead (to eliminate the influence of holiday mobility on *R_t_*), the relative reduction in the *R_t_* was estimated to be 0·20 (0·13, 0·27) in Hokkaido, 0·50 (0·43, 0·57) in Gifu, and −0·01 (−0·25, 0·21) in Mie. Similarly, compared with the 14-day period before the holiday began, the relative reduction in *R_t_* was estimated to be 0·23 (0·18, 0·28) in Hokkaido, 0·47 (0·41, 0·53) in Gifu, and 0·08 (−0·06, 0·21) in Mie.
