# Supplementary material for: Assessing Public Health and Social Measures Against COVID-19 in Japan From March to June 2021
Source: Front Med (Lausanne). 2022 Jul 12;9:937732. doi: 10.3389/fmed.2022.937732 (PMC9315273; doi:10.3389/fmed.2022.937732)
Supplement: Supplementary file 5 [file Table_2.docx]

**Table S2 Reduction in the effective reproduction number (Rt) comparing a varying baseline period with the entire state of emergency (SoE) period**

| Prefecture | Absolute reduction in Rt (7 days pre-SoE vs entire SoE period) | Relative reduction in Rt (7 days pre-SoE vs entire SoE period) | Absolute reduction in Rt (14 days pre-SoE vs entire SoE period) | Relative reduction in Rt (14 days pre-SoE vs entire SoE period) |
| --- | --- | --- | --- | --- |
| Hokkaido* | 0.67 (0.61, 0.75) | 0.47 (0.44, 0.50) | 0.68 (0.63, 0.74) | 0.47 (0.45, 0.50) |
| Tokyo | 0.22 (0.17, 0.28) | 0.20 (0.16, 0.23) | 0.24 (0.21, 0.28) | 0.21 (0.18, 0.23) |
| Aichi | 0.34 (0.25, 0.42) | 0.29 (0.23, 0.35) | 0.40 (0.33, 0.47) | 0.33 (0.28, 0.38) |
| Kyoto | 0.46 (0.33, 0.59) | 0.35 (0.28, 0.41) | 0.47 (0.39, 0.55) | 0.35 (0.31, 0.40) |
| Osaka | 0.65 (0.61, 0.70) | 0.47 (0.45, 0.49) | 0.74 (0.70, 0.77) | 0.50 (0.48, 0.51) |
| Hyogo | 0.79 (0.71, 0.88) | 0.53 (0.50, 0.56) | 0.81 (0.74, 0.87) | 0.53 (0.51, 0.55) |
| Okayama | -0.07 (-0.20, 0.08) | -0.16 (-0.50, 0.15) | 0.60 (0.50, 0.71) | 0.61 (0.52, 0.70) |
| Hiroshima | 0.41 (0.31, 0.53) | 0.41 (0.32, 0.50) | 0.67 (0.60, 0.75) | 0.55 (0.50, 0.60) |
| Fukuoka | 0.53 (0.47, 0.60) | 0.49 (0.44, 0.53) | 0.56 (0.51, 0.62) | 0.50 (0.47, 0.54) |
| Okinawa | -0.28 (-0.43, -0.11) | -0.28 (-0.47, -0.10) | -0.20 (-0.33, -0.07) | -0.18 (-0.32, -0.06) |

Values in parentheses are bootstrapped 95% confidence intervals.

* The 7- and 14-day pre-SoE baseline period in Hokkaido overlapped with a 10-day national holiday. Using a 7-day baseline period before the start of the holiday period instead (to eliminate the influence of holiday mobility on *Rt*) the relative reduction in *Rt* was estimated to be 0·47 (0·43, 0·52). Similarly, using a 14-day period before the holiday began, the relative reduction in *R_t_* was estimated to be 0·50 (0·46, 0·53).
