# Supplementary material for: Assessing Public Health and Social Measures Against COVID-19 in Japan From March to June 2021
Source: Front Med (Lausanne). 2022 Jul 12;9:937732. doi: 10.3389/fmed.2022.937732 (PMC9315273; doi:10.3389/fmed.2022.937732)
Supplement: Supplementary file 6 [file Table_3.docx]

**Table S3. Comparison of Rt of 7 days before, 14 to 8 days before and 7 days after PEM**

| Prefecture | Average Rt during the 14-8 days pre- PEM declaration | Average Rt during the 7 days pre- PEM declaration | Average Rt during the 7 days post-PEM declaration |
| --- | --- | --- | --- |
| Hokkaido | 1.45 | 1.43 | 1.09 |
| Tokyo | 1.10 | 1.19 | 1.15 |
| Aichi | 1.26 | 1.21 | 1.09 |
| Kyoto | 1.28 | 1.30 | 1.24 |
| Osaka | 1.58 | 1.31 | 1.27 |
| Hyogo | 1.47 | 1.43 | 1.29 |
| Okinawa | 1.45 | 1.43 | 1.09 |
| Miyagi | 1.10 | 1.19 | 1.15 |
| Chiba | 1.26 | 1.21 | 1.09 |
| Saitama | 1.28 | 1.30 | 1.24 |
| Ehime | 1.58 | 1.31 | 1.27 |
| Mie | 1.47 | 1.43 | 1.29 |
| Gifu | 1.07 | 1.17 | 0.91 |
| Okayama | 0.78 | 1.24 | 0.90 |
| Hiroshima | 1.29 | 1.07 | 1.10 |
| Gunma | 1.25 | 1.21 | 1.08 |
| Ishikawa | 1.07 | 0.71 | 0.64 |
| Kumamoto | 0.69 | 1.18 | 0.83 |
